# Medical pluralism and livestock health: ethnomedical and biomedical veterinary knowledge among East African agropastoralists

**DOI:** 10.1186/s13002-017-0135-1

**Published:** 2017-01-21

**Authors:** Mark A. Caudell, Marsha B. Quinlan, Robert J. Quinlan, Douglas R. Call

**Affiliations:** 1 Paul G. Allen School for Global Animal Health, Washington State University, Pullman, WA USA; 2 Department of Anthropology, Washington State University, Pullman, WA USA; 3 Nelson Mandela African Institution of Science and Technology, Arusha, Tanzania

**Keywords:** Ethnoveterinary medicine, Medical pluralism, Cultural consensus, One Health, Agropastoralists, East Africa, Tanzania, Ethiopia

## Abstract

**Background:**

Human and animal health are deeply intertwined in livestock dependent areas. Livestock health contributes to food security and can influence human health through the transmission of zoonotic diseases. In low-income countries diagnosis and treatment of livestock diseases is often carried out by household members who draw upon both ethnoveterinary medicine (EVM) and contemporary veterinary biomedicine (VB). Expertise in these knowledge bases, along with their coexistence, informs treatment and thus ultimately impacts animal and human health. The aim of the current study was to determine how socio-cultural and ecological differences within and between two livestock-keeping populations, the Maasai of northern Tanzania and Koore of southwest Ethiopia, impact expertise in EVM and VB and coexistence of the two knowledge bases.

**Methods:**

An ethnoveterinary research project was conducted to examine dimensions of EVM and VB knowledge among the Maasai (*N* = 142 households) and the Koore (*N* = 100). Cultural consensus methods were used to quantify expertise and the level of agreement on EVM and VB knowledge. Ordinary least squares regression was used to model patterns of expertise and consensus across groups and to examine associations between knowledge and demographic/sociocultural attributes.

**Results:**

Maasai and Koore informants displayed high consensus on EVM but only the Koore displayed consensus on VB knowledge. EVM expertise in the Koore varied across gender, herd size, and level of VB expertise. EVM expertise was highest in the Maasai but was only associated with age. The only factor associated with VB expertise was EVM expertise in the Koore.

**Conclusions:**

Variation in consensus and the correlates of expertise across the Maassi and the Koore are likely related to differences in the cultural transmission of EVM and VB knowledge. Transmission dynamics are established by the integration of livestock within the socioecological systems of the Maasai and Koore and culture historical experiences with livestock disease. Consideration of the nature and coexistence of EVM and VB provides insight into the capacity of groups to cope with disease outbreaks, pharmaceutical use patterns, and the development of community health interventions.

**Electronic supplementary material:**

The online version of this article (doi:10.1186/s13002-017-0135-1) contains supplementary material, which is available to authorized users.

## Background

Animal husbandry systems are an expanding sector of agricultural production in low-income countries where they are driven by increasing global demand for meat and milk, and climatic changes unfavorable to crop production [1–3]. Animal disease is a major constraint on the productivity of these systems. Residents of low-income countries often approach livestock disease treatment using ethnoveterinary medicine (EVM), which encompasses indigenous or “traditional” beliefs, knowledge, skills and practices pertaining to the healthcare of animals [4, 5]. The majority of these societies also rely upon “biomedicine,” defined as the global medical system based upon western scientific principles and products (e.g., antibiotics) [6–9]. Reliance on veterinary biomedicine (VB) has received little anthropological attention [10]. The coexistence of EVM and VB patterns health outcomes in both livestock and human populations. Replacement of a traditional plant remedy with pharmaceutical antibiotics, for example, may initially decrease livestock mortality, but can contribute to increased selection for antimicrobial resistance [11]. Higher livestock mortality, in turn, can increase human morbidity and mortality by reducing consumption and household income from livestock sales.

Individual and sociocultural factors frame the transmission of veterinary knowledge. Culture-level framing includes differences in livestock subsistence practices, role of animal health professionals, and the history and ecology of livestock disease. Individual-level framing includes engagement in the livestock sector and personal illness experience. These factors likely interact in a dynamic fashion to shape health outcomes and knowledge transmission. To examine these dynamics, we compare patterns of expertise (often termed “cultural competence”) and cultural consensus (i.e., agreement across a group) in EVM and VB within two East African agropastoral populations, the Maasai of northern Tanzania and the Koore of southern Ethiopia. The Maasai and the Koore provide an informative comparison on the coexistence of EVM and VB given differences in herd sizes and composition, the economic and social contributions of livestock, and local histories and ecologies of livestock diseases.

## The coexistence of traditional medicine and biomedicine

Medical pluralism, where traditional medicine is practiced alongside biomedicine, has a long history of overlap in indigenous populations within low-income countries [12–18]. How these “two medicines” coexist remains a matter of debate. Evidence indicates that the relationship can be complementary [14, 17, 19–24] or competitive [25–27]. For example, Giovannini et. al [28] found that medicinal plant knowledge was significantly and positively associated with pharmaceutical knowledge in an indigenous Mexican community. In contrast, Vandebroek et al. [26] found that knowledge and use of medicinal plants negatively correlated with pharmaceutical use in the Bolivian Amazon. Even if biomedicines replace traditional treatments, people may still draw on traditional knowledge to inform diagnoses, thereby framing treatment options [29]. Incorporation of both ethnomedicine and biomedicine emphasizes the need to identify the factors that drive healing competence, adoptions of new treatments, and maintenance of traditional treatments within evolving indigenous medical systems.

### Correlates of expertise

Research on the correlates of traditional medical expertise has largely emphasized ethnobotanical knowledge of plants used to treat human conditions [26, 28, 30–33] although the same plants may be used to treat livestock [34–40]. Expertise is associated with demographic dimensions in ways consistent with the gendered and age-based division of labor often observed in many small-scale societies [39, 41]. For example, where men tend to work with animals, older women know more about plants while men tend to know more about animal behavior [28, 42–45]. Children tend to become competent in ethnobotany by the pre-teen years [46, 47]. Unlike subsistence and other ethnobotany knowledge, adult medical ethnobotany skill often increases with age [33, 42, 48, 49]. The effect of “modernity” on ethnomedical knowledge is ambiguous. Education can be negatively [50–53] and positively correlated with expertise [54, 55], while others have found no relationship [44, 56]. Other indicators of modernity (e.g., cash earnings) associate with greater medicinal competence in Dominica [33], but appear unrelated to variation in expertise among Tsimane horticulturalists in Bolivia [54].

Whether correlates of expertise in ethnobotanical/ethnomedical knowledge are associated with ethnoveterinary expertise remains largely unclear. Nevertheless, livestock-keeping populations hold extensive knowledge of livestock disease prevention, diagnosis, and both traditional and novel biomedical treatments [7, 35, 38, 40, 57–61]. Studies quantifying expertise and cultural consensus have documented high levels with respect to EVM [38, 62] but lower levels for VB [63]. Among Kikuyu farmers of Kenya, high consensus was found for what plants were used to treat anaplasmosis, East Coast Fever and ectoparasites [38]. In contrast, Fulbe pastoralists displayed a lack of knowledge on “Western” disease concepts, specifically, whether livestock diseases can be zoonotic [63]. Most ethnoveterinary research has gathered data from “experts” (e.g., traditional healers) meaning there is little variation in which the correlates of expertise can be examined [8, 64]. Studies examining more demographically diverse samples have documented variability in ethnoveterinary knowledge. Among the Nu people of China, gender-based variability in ethnoveterinary knowledge are consistent with division of labor differences [41]. Informants in other studies have claimed that children who attend school have less traditional veterinary knowledge [63], which is consistent with some findings from ethnobotany as well as a recent study of Maasai students [65].

Levels of expertise in EVM and VB impact small-holder livelihoods given direct links to both livestock and human health outcomes. Expertise in EVM remains important for livestock health, particularly in communities with limited access (money or availability) to biomedicine [8, 35, 59]. Pharmacological research provides evidence that traditional remedies can be effective against a number of common diseases, including African sleeping sickness (*Trypanosomiasis*) [66], helminthic infections [67], skin infections [68], and can exhibit antimicrobial properties [67]. Expertise in VB is important given that veterinary pharmaceuticals substantially reduce livestock mortality rates and by promoting prudent antibiotic use may preserve the long term efficacy of drugs by limiting development of antimicrobial resistance [11, 69]. Non-prudent drug usage is particularly concerning in low-income countries where pharmaceutical use often occurs outside a professional veterinary context [35, 70]. Nevertheless, it is believed that many more animals die due to a lack of access to antimicrobials compared to infections caused by resistance bacteria [35]. To provide insight into the potential factors impacting livestock treatments within our study populations we next discuss the contexts of EVM and VB in the Maasai and the Koore.

## Study populations

### The Maasai

The Maasai and related *Maa*-speaking pastoralists are found throughout Tanzania and Kenya [71, 72]. Our study was conducted among Maasai living in Nandonjukin, a rural village within Simanjiro District in the Manyara Region in northern Tanzania. Maasai were traditionally nomadic pastoralists who moved with their herds in search of forage and water. Today most Maasai are agropastoralists who grow crops (mostly maize and beans) although livestock products remain staples, particularly cattle milk and meat from goats/sheep [73]. Cattle milk and milk products (e.g., butter) contribute between a third to half of the energy in Maasai diets [74]. Maasai traditionally measure wealth by the cattle herd size and usually pay bride price in livestock. Livestock are investments and savings, and their sale provides a major income source that is increasingly important given the necessity of cash for schooling and healthcare. Livestock are also of symbolic importance, particularly cattle milk, which is integrated into age-set ceremonies and used to confer blessings [75, 76]

The diverse roles of livestock ensure that EVM and VB remain vital for the sustainability of Maasai livelihoods. Most Maasai still diagnose and treat livestock diseases themselves, largely outside of consultation with veterinary professionals [6, 77, 78]. Maasai diagnosis is primarily symptom-based (e.g., piloerection, panting, lethargy, or loss of appetite) although they consider the present season, age, sex, and species affected, forage location, and disease information from local herds [6, 77]. Diagnosis and treatment is usually carried out by the man who owns the livestock but is often done in cooperation with other Maasai men, who either live within or are visiting the *nkang* (an extended family compound). Less frequently, owners confer with livestock extension officers and veterinary drug shop owners. Herder boys, who begin tending livestock by the ages of 5–6, recognize general disease symptoms, such as piloerection (*isuuto* in *Maa*) and will notify their fathers of sick animals after returning with the herds in the evening. Women and girls, who are responsible for milking, also notice diseases, especially if the animal’s milk production decreases.

Maasai rely on both traditional medicine and biomedicine to treat their animals. Maasai believe that trees and bushes are medicinal and their word *olcani* (singular) means both “tree” and “medicine” [79]. When Maasai refer to medicine generally or to Maasai ethnomedicine specifically, they use *olcani*. The Maasai have eagerly adopted pharmaceutical veterinary medicines, including vaccines, antimicrobials, and insecticide dips, which they refer to as “exotic medicines.” Maasai purchase these medicines at special veterinary drug shops, existing even in the smallest villages, or if medicines are inaccessible due to shop availability or cash limitations, people also borrow them from family/friends [80]. Drug shops are licensed to certified animal health specialists, but, on any day, a clerk with no specialized training may be the only attendant. Qualitative interviews and observation studies suggests Maasai livestock owners are generally aware of the recommended course of treatment and dosage practices, but may give the same dosage regardless of weight, which could lead to both under and overdosing [6, 80]. Following best-practices for pharmaceuticals, especially antimicrobials, is important for Maasai health as they rarely observed withdrawal periods when treating animals [6].

### The Koore

The majority of Koore live in the Amaro Zone of the Southern Nations, Nationalities, and Peoples’ Region in southwestern Ethiopia [81, 82]. The current study was conducted in Gamule, a lowland *kebele* outside of Kelle, the capital of the Amaro Zone. The Koore cultivate areas of the Amaro Mountain range and have more recently migrated into the lowlands of the Western Rift Valley [83]. Primary staple crops include ensete (*Ensete ventricosum*), grains (e.g., maize and teff), legumes, and bananas while coffee and chat are grown as cash crops. The cultural history of livestock integration into Koore livelihoods is more variable compared to the Maasai. Historically, cattle were largely kept for milk and average herd sizes were likely smaller compared to the herds kept by lowland Koore today [83]. As the Koore incorporated grains into their livelihoods, particularly teff, they began to keep cattle for draught power. Livestock are also essential as a source of fertilizer for enset, which does not grow well without manure inputs [84]. Recent migration into the lowlands has meant that more Koore have begun keeping livestock for sale. Keeping livestock for sale has increased herd sizes, which were likely relatively smaller in the past given limited pastureland in the highlands and later use as draught power (Awoke Assoma, personal communication). Indeed, in the current study over 65% of households had increased livestock herd size in the last 5 years. The average Koore herd (28 head) is still about 14 times smaller than the average Maasai herd (429 head). If herd size correlates with contagious disease incidence, we should expect the Koore to have less experience with disease.

Economic and direct dietary dependence on livestock is considerably less within the Koore compared to the Maasai. One average, sales of livestock and livestock products contributed about one-fifth to Koore household income compared to over half for the Maasai, although this obscures the contribution of livestock as draught power and fertilizer. Koore also consume fewer livestock products than the Maasai with an average of 5% eating meat more than once a month compared to an average of 65% in the Maasai. Additionally, the average Koore household consumes 77% less milk than the average Maasai household. Contributions of livestock in lowland Koore livelihoods may be curtailed given the uncertainty associated with the relatively new strategy of selling livestock. In the current sample, a majority of Koore had lost one-half or more of their herds to disease or drought within the last 5 years while only one-fifth of Maasai had lost half or more of their herd across the same period.

Koore traditional ethnoveterinary knowledge has received less attention compared to the Maasai (but see, [85]). Like the Maasai, Koore men usually diagnose and treat animals themselves. Koore men are more likely, however, to seek out professional consultation, either with Community Animal Health Workers (CAHWS) or the proprietor of the local veterinary drug shop, who runs the shop himself and has his Doctorate of Veterinary Medicine (DVM). The DVM stated that many farmers will bring their sick animals directly to his shop (almost unheard of in the Maasai), where he will diagnose the disease. He offers to show the farmer how to prepare the solution, the appropriate injection site, and provides information on the appropriate dosage. Gender differences also exist between the Maasai and Koore. While Koore women and girls milk cows over 27% of Koore households had no milking livestock compared to 1% in the Maasai, which may mean Koore females are less likely to interact with livestock. The Koore level of market integration and education also contrasts with the Maasai. Koore children are more likely to attend school (Maasai ≈ 40% vs Koore ≈ 80%) so Koore children may have fewer interactions with livestock. Koore were also more likely to diversify their livelihoods with income from self-employment, wage, or salary labor. See Table 1 in Method’s section for key livelihood differences in the Maasai and Koore.

**Table 1:** Summary Statistics for Maasai and Koore

	Maasai (*N* = 146)				Koore (*N* = 100)			
	Mean	SD	Min	Max	Mean	SD	Min	Max
Sex (1 = Male 0 = Female)	0.53	0.50	0	1	0.60	0.43	0	1
Age	33.19	20.09	7	100	26.86	16.16	9	85
Children^a^	7.52	2.82	0	22	7.45	2.74	0	17
Education level	1.46	0.91	1	7	2.12	3.30	1	20
Total Cattle^a^	104.48	218.43	0	1400	16.66	12.19	2	44
Total Small-Stock^a^	179.09	275.90	0	1200	8.38	8.97	0	37
Standardized	0	1	−.0.66	3.30	0	1	−1.28	2.06
Income types^a^	0.39	0.65	0	2	0.70	0.26	0	3
Seek Professionals^a^	1.05	0.94	0	4	2.22	0.53	1	3
Total Income per month^a^	53.26	835.19	0	2730.50	7.75	10.81	0	21.91
Livestock Sickness^a^	0.89	0.32	0	1	0.90	0.31	0	1
Days away due to sickness^a^	2.36	1.09	1	7	1.59	1.05	1	4
VB Expertise	84.16	9.62	32	100	93.18	6.05	69	100
EVM Expertise	77.66	14.51	32	100	60.01	11.93	34	85

^a^Data was collected from household heads only

### Predictions

Considering the breadth of research on the correlates of ethnoveterinary expertise and the context of livestock, VB, and EVM in Maasai and Koore livelihoods, we derive the following predictions.

1. If EVM and VB are competitive knowledge bases then those competent in EVM should be less competent in VB and vice versa. If complementary, so that one informs the other, then those competent in EVM should be competent in VB and vice versa
2. If education decreases EVM as with other ethnomedical domains we should expect expertise to be lower for those individuals with more years of schooling and, across groups, lower in the Koore compared to the Maasai.
3. If gendered division of labor impacts EVM and VB we should expect women in both groups to have lower expertise than men. Koore women should know less compared to Maasai women.
4. If market integration decreases EVM, we should expect individuals with more diversified livelihoods to be less competent and, across groups, the Koore should have less expertise than the Maasai
5. If consultation with professional veterinary services promotes use of pharmaceutical veterinary medicines and transmission of VB knowledge we should expect individuals who are more reliant on professional vets and livestock extension officers to be more competent in VB and the Koore to be more competent than the Maasai.

## Methods

### Survey development

Several substantive and practical considerations led us to compare the Maasai and the Koore. First, as discussed above, the Maasai and the Koore vary in their past and present reliance on livestock and one of our interests was determining how cultural, economic, and historical differences impact EVM and VB. Second, the Maasai and Koore, to the author’s knowledge, have no history of interaction (e.g., migration between two groups) that could impact the distribution of EVM and VB between the two groups through knowledge transmission. Third, and more practically, we have been working alongside both populations on various projects since 2012. Due to this sustained relationship, we have established rapport with community members and well-trained assistants are available to aid in project development and management.

Survey development was guided by the “ethnographic funnel,” which proceeds from focus group and key informant interviews to the construction of survey components [86–88]. Domain analysis of ethnoveterinary medicine was conducted by first consulting community members to identify livestock disease experts. These key informants were asked to free-list all livestock diseases. Successive free-list prompts were used to identify symptoms, causes, treatments, and local histories [89, 90]. Contrast verification questions were used to identify characteristics unique to each recalled disease (e.g., The first symptom of Disease A is not the first symptom of disease B, C, D, E…etc.) [88]. Results were used to construct 30–40 item EVM questionnaires. Items were multiple choice (4 potential answers) and balanced between positively and negatively worded questions. The questionnaire elicited information on only ten diseases although informants listed more diseases (Maasai = 22, Koore = 16) (see Additional file 1 for a list of diseases and logic for inclusion). The VB questionnaire covered three different pharmaceuticals (see Additional file 1 for list of medicines). For each medicine, questions on the correct dosage and treatment periods for 1) large cattle, 2) small cattle, 3) calves, and 4) small stock were included (eight questions per medicine = 24 questions total). Questions were multiple choice with five items for correct dosage (cubic centimeters) and four items for treatment periods (days). Back translation was used to ensure the correct translation of survey items into Swahili (Tanzania) or Amharic (Ethiopia). A field crew of four Maasai assistants and three Koore assistants were given 1–2 weeks training.

### Sampling

A census was conducted in both study communities to generate a random sample. All homes within Gamule *Kebele* were visited and the names of all parents recorded. In Nadonjukin, *bomas* accessible by vehicle were visited and all parents were recorded. From the list of adults (Koore:94 Maasai:175), 50 names were randomly selected for subsequent interviews. Children were selected for interview by alternating between the criteria of age (preteen vs. teen) and gender (sons vs. daughters). If a child who met the criteria was not available, the next pair of inclusion criteria was used. All interviews were conducted in private and out of hearing of other household inhabitants. Informants were asked not to discuss the survey until we had completed our work. Tanzania informants were paid 10,000 *shilingi* (5000 for children) and Ethiopian informants 30 *birr* (15 for children).

### Variables

Summary statistics are provided in Table 1. “Livestock sickness” is the average number of days per month that caring for sick livestock deducts from other activities (scaled 1 = 1–2 days, 2 = 3–6 days, 3 = 7–14 days, 4 = 15+ days). “Seek professionals” is a scale (1–3) reflecting reliance on veterinary professionals (consulted veterinarian, livestock extension officer, or visited drug shop). “Income types” is a scale of diversification (1–3), including sources of income from self-employment, wage labor, and salary labor. Education is coded 1 = no formal, 2 = some primary school, 3 = completed primary school, 4 = some secondary school, 5 = completed secondary school, 6 = some college, 7 = finished college. To facilitate the comparison of how income impacts EVM and VB across the Maasai and the Koore, total income was converted from the Tanzania *shilingi* and Ethiopian *birr* to US Dollars. “VB Expertise” is an individual’s score out of 100 on the VB questionnaire. “EVM Expertise” is an individual’s score on the EVM questionnaire and is out of 100.

### Consensus and measures of expertise

Expertise in EVM and VB was calculated by determining the correct responses based upon answer keys. The answer key for EVM came from responses by traditional experts in focus group and key informant interviews. The answer key for VB was taken from relevant manufacturer websites on the recommended dosage and treatment periods [91, 92]. Cultural consensus was quantified using the formal consensus model in UCINET [93].

### Analysis

Ordinary least-squares regression was used for all analyses. All non-dichotomous variables or those not already standardized were mean centered. Koore is the reference ethnicity for all models (Koore = 0, Maasai = 1). For analysis, household level information was applied to all household members (e.g., a child was associated with his/her father’s herd size). Interactions by ethnic group were specified for all variables and were removed if neither the main effect or interaction was significant. See Additional file 1 for further discussion on model diagnostics and fit.

## Results

### Consensus in EVM and VB

High levels of consensus were found on EVM knowledge for both groups, although the Maasai exhibited higher consensus compared to the Koore (ratio of the largest to second largest eigen value for Maasai = 17.98, Koore = 11.2). Consensus on VB knowledge was high among the Koore (eigen value ratio = 14.5) while there was no consensus among the Maasai (eigen value ratio < 3) (Fig. 2).

### Predictors of traditional expertise

**Fig. 1:**
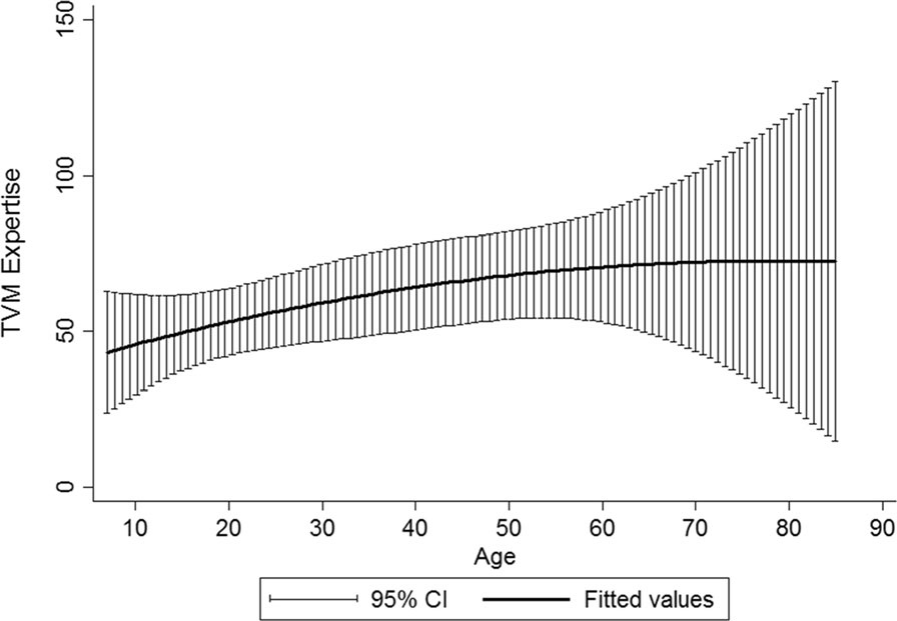
EVM expertise across age in the Koore

**Fig. 2:**
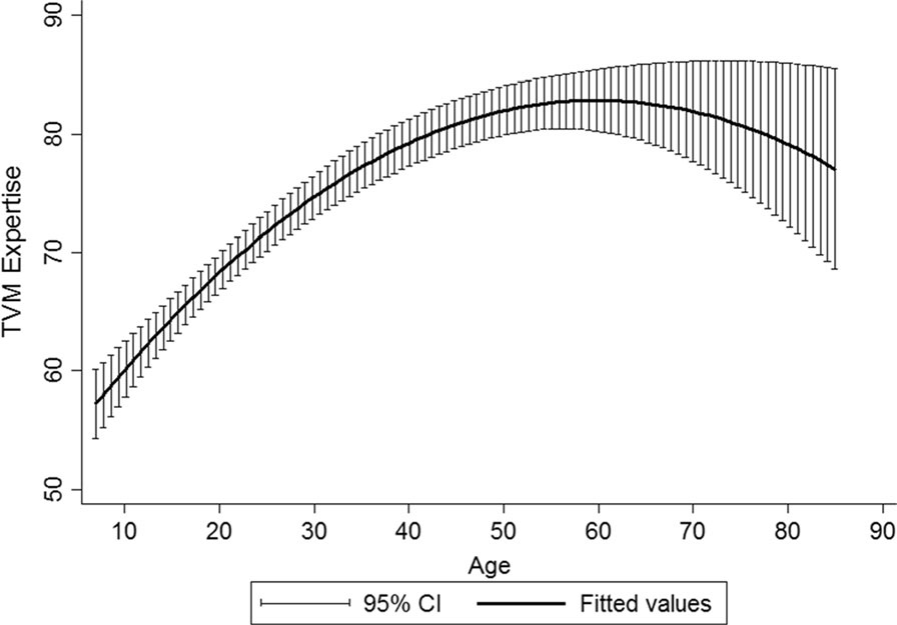
EVM expertise across age in the Maasai

Demographic and socioeconomic attributes accounted for about 45% of the variance in EVM in the Koore and 29% in the Maasai (Table 2). Koore females were predicted to score 54 on the EVM questionnaire at the mean of herd size, VB knowledge, age, education, and integration with market economy and professional veterinary services. Koore males had significantly higher EVM scores, averaging around 63. Herd size was positively associated with EVM expertise with every additional animal increasing the score by 0.132 points. Every additional point on the VB expertise questionnaire predicted a 0.598 increase on the EVM expertise. Age showed a weak quadratic relationship with expertise, with EVM increasing and then leveling off around 70 years of age, although confidence intervals increased at higher ages (Figs. 1 and 2). No other factors including education level, livelihood diversification, and consultation with professional livestock health workers were significantly related to EVM expertise. In the Maasai, females were predicted to score 82 on the EVM questionnaire, which was not significantly different than the predicted score for Maasai males. The sole attribute significantly related to EVM in the Maasai was age, with expertise increasing until the age 60 and then decreasing.

**Table 2:** Correlates of traditional expertise in the Maasai and Koore

	Koore		Maasai	
	*B*	95% CI	*B*	95% CI
Herd Size	0.132	0.014–0.251*	0.001	−0.002–0.004
VB Expertise	0.598	0.150–1.046**	−0.000	−0.189–0.189
Sex	9.209	2.376–16.041**	−0.935	−7.599–5.730
Age	0.463	0.316–0.610**	0.429	0.287–0.572**
Age Sqrd	−0.013	−0.019– −0.006**	−0.008	−0.013– −0.002**
Education	−0.303	−2.196–1.591	−0.051	−2.355–2.252
Income Types	−0.108	−10.334–10.118	2.003	−1.456–5.463
Professional Help	0.182	−4.419–4.784	−0.639	−3.128–1.850
Constant	53.734	45.538–61.931**	82.314	75.487–89.141**
Adjusted *R* ^2^	0.45		0.29	
N	98		114	

Females are the reference group. VB expertise is the score from the biomedical questionnaire

*p < 0.05; **p < 0.01

### Predictors of biomedical expertise

Demographic and socioeconomic attributes accounted for about 16% of the variance in VB in the Koore and 0.2% in the Maasai (Table 3). Koore females averaged a score of 85.8, which was not significantly different from Koore males 85.7. The only attribute significantly related to VB expertise in the Koore was EVM expertise with a one-point increase associated with a 0.15 point increase in VB expertise. Maasai females averaged a VB score of 78.8, which was not significantly different than the Maasai males (81.6). No demographic or socioeconomic attributes were significantly related to biomedical expertise in the Maasai.

**Table 3:** Correlates of biomedical expertise across the Maasai and Koore

	Koore		Maasai	
	*B*	95% CI	*B*	95% CI
Herd Size	0.016	−0.047–0.078	−0.002	−0.005–0.001
EVM Expertise	15.585	3.912–27.259**	−0.016	−19.859–19.826
Sex	−0.157	−3.823–3.508	2.835	−3.980–9.650
Age	−0.018	−0.112–0.076	0.010	−0.160–0.179
Age Sqrd	0.000	−0.003–0.004	0.003	−0.002–0.009
Education	−0.315	−1.279–0.649	1.394	−0.953–3.740
Income Types	−0.088	−5.308–5.132	2.284	−1.258–5.826
Professional Help	−0.454	−2.800–1.893	−0.542	−3.096–2.011
Constant	85.829	81.581–89.076**	78.861	71.700–86.023**
Adjusted R2	0.16		0.002	
*N*	98		114	

Koore and female are the reference group (i.e., Koore = 0 and female = 0)

## Discussion

We examined two dimensions of Maasai and Koore ethnomedicine motivated by etic categories based on an assumed opposition of traditional vs biomedical knowledge. Consistent with their greater economic, social, and spiritual reliance on livestock, the Maasai showed higher levels of expertise in EVM compared to the Koore (82 versus 60), although both groups displayed consensus. In general, these findings support previous work documenting high levels of consensus on EVM in smallholder societies [38, 62] It was the Koore, however, who had greater VB expertise and also consensus on VB. This result is particularly surprising given the Maasai, due to differences in herd sizes and local disease ecology, should be more frequent users of veterinary pharmaceuticals. It may be that the lower levels of expertise and consensus on EVM in the Koore facilitated the adoption of VB. Lower expertise likely makes traditional treatment outcomes more variable while lower consensus makes diagnosis and decisions on the “best treatments” more uncertain across a group. This uncertainty may have pressured Koore to seek out and accept knowledge from professional livestock health workers, thereby promoting the horizontal (peer) and oblique (older peer) transmission of VB knowledge. Theoretically, horizontal and oblique transmission can result in high levels of cultural consensus and rapid cultural change when compared to vertical transmission (parent to child) [94, 95]. Rapid cultural change may have been selected for as the Koore descended into the lowlands, expanded their herds, and experienced more livestock disease. Understanding the transmission dynamics of EVM and VB knowledge will be important because *who* transmits information and *how* information is transmitted (e.g., teaching, casual communication or observation) impacts expertise and consensus and thus a population’s ability to respond to disease outbreaks and adopt new health innovations.

Another group-contrast was that more individual attributes were related to EVM expertise in the Koore compared to the Maasai (Tables 2 and 3). Consistent with studies finding differences in expertise across gender roles [41, 44, 45], Koore males had higher EVM expertise compared to Koore females. In both populations and in support of earlier studies [33, 42, 43] age demonstrated a positive relationship to EVM. However, this relationship held only until the age of about 65, after which the relationship between EVM and age started to decrease. These associations with individual attributes may reflect how differing cultural histories of livestock disease impact the dynamics of knowledge transmission. Partially, these differing histories are due to the prevalence of livestock disease in areas traditionally inhabited by the Maasai and the Koore as well as the movement of livestock across the landscape. The Maasai inhabit warmer lowlands areas where common parasitic vectors of livestock disease (*Trypanosoma congolense*) are more common in comparison to the cooler highland environments traditionally favored by the Koore [96, 97]. Maasai transhumance further increases interactions among and between livestock and wildlife herds, elevating the risk of disease transmission [98, 99]. Indeed, high rates of livestock mortality have been documented in many pastoral groups and the Maasai specifically, whose herds were decimated (up to 80% mortality rate) from diseases that swept the Maasai Steppe (e.g., Rinderpest) in the late 19th and early 20th centuries [71]. Persistent disease threats may have ensured that EVM became an important part of the “mechanical solidarity” [100] of Maasai culture, and was thereby transmitted to all group members. This pattern of transmission is consistent with the high levels of consensus and expertise displayed in the Maasai and may underlie the lack of association with gender (prediction 2), education levels (prediction 3), market integration (prediction 4), or interaction with veterinary professionals (prediction 5).

In contrast to the Maasai, the smaller and less mobile herds of the Koore may have been insulated from disease in the highlands of the Amaro Range. Although historical data is not available for the Koore, informants were adamant that livestock disease was more prevalent in the lowlands. Lower disease frequencies, combined with the less central role of livestock within Koore livelihoods, may have minimized selection for the widespread integration of EVM into Koore culture. Consequently, the evolution and transmission of EVM was more likely to be a function of individual experience among the Koore, which seems to be supported by our results finding that herd size and gender were significantly associated with EVM expertise in the Koore. Finally, it was only in the Koore that VB expertise displayed a complementary relationship with EVM expertise. Such complementarity is consistent with some ethnobotanical studies [14, 17, 28] and we do not find evidence that EVM and VB are competing knowledge bases [26]. A recent study among the Maasai, however, did find that reliance on traditional healers and lay treatment of antibiotics were negatively associated with use of professional veterinarians [6].

## Future directions and limitations

Limitations associated with the current study point to productive avenues for future research. Imposition of two etic dimensions may be appropriate to assess avenues for public health communication but an alternative “grounded theory” approach might be equally useful. Future analyses might explore the structure of pooled items from EVM and VB scales to reveal dimensions emerging from Maasai and Koore patterns of veterinary practice. We might find, for example, that facets of both traditional and biomedical knowledge cluster in medical syncretism reflecting local adaptation.

The likely role of cultural-histories, disease ecologies, and livelihoods in the patterning of ethnomedical/ethnobiological knowledge highlights the need for systems-based perspectives in understanding disease response in small-scale societies. Perspectives such as socioecological systems theory [101] will be instrumental given ongoing changes in the economic, social, and ecological spheres that are multivariate, nonlinear, and generate complex feedback loops [73, 102]. These changes, including decreasing levels of transhumance and an increasing reliance on crop cultivation, may impact the distribution of knowledge. For example, as livelihoods shift towards greater emphasis on crop cultivation EVM knowledge may become “decoupled” from mechanical solidarity of groups and more linked to individual experience. A counterbalance to this trend could be increasing reliance on livestock-keeping due to uncertainties of climate change [1], which may ensure knowledge is transmitted to most members within a society.

Methodologically, variables meant to quantify integration with market-economy and the professional veterinary sector were scales based upon yes-no responses (e.g., did you visit a vet in the last 6 months?). These scales only measure the existence and not the extent of integration. If extent was better operationalized we may have discovered effects of modernity variables on EVM and VB expertise. However, this the lack of association between modernity and expertise is consistent with earlier findings [44, 56]. Second, group differences in VB expertise and consensus could be an artifact of the VB questionnaire. For example, we may expect differences if the Maasai have access to a greater variety of pharmaceuticals and individuals vary in their favorite medicines (e.g., show brand loyalty), while the Koore are forced to use the same 3–4 medicines every time. Alternatively, medicines used to quantify VB expertise might vary in their histories of use within groups. Where possible, future studies should consider gathering drug-specific data, including aspects of the pharmacopeia, when a particular drug was introduced into a community, and by whom it was introduced by (e.g., government program, word of mouth).

## Conclusions

Livestock-dependent populations in low-income countries continue to diagnose and treat livestock diseases by themselves. Self-treatment ensures that the traditional ethnoveterinary and biomedical knowledge bases within these groups are linked with herd productivity and so ultimately livelihood security. Here we showed that EVM and VB coexist differently, are associated with different individual attributes, and display varying levels of consensus across two East African agropastoral groups, the Maasai of Tanzania and the Koore of Ethiopia. Compared to the Koore, the Maasai exhibited higher consensus and expertise on EVM. In contrast, the Koore had higher expertise in VB knowledge and, unlike the Maasai, displayed consensus on this knowledge base. Further, it was only among the Koore that expertise in EVM and VB were positively associated. We argue these variations in expertise, consensus, and patterns of coexistence between the two knowledge bases reflect differences in the culture-history of animal husbandry and the ecologies of disease within each group. Future work should examine how differences in transmission impact the distribution and coexistence of EVM and VB. Expansion of this research to include more livestock-dependent populations will be important to understand (quantitatively) how cultural differences impact EVM and VB. Considerations of these effects will be necessary to develop approaches to respond to future disease outbreaks, maintain the efficacy of pharmaceutical drugs, and aid efforts to reduce the emergence and evolution of antimicrobial resistance.

## Additional file

Additional file 1: Surveyed Diseases and Model Diagnostics. (DOCX 669 kb)
