# Supplementary material for: Medical pluralism and livestock health: ethnomedical and biomedical veterinary knowledge among East African agropastoralists
Source: J Ethnobiol Ethnomed. 2017 Jan 21;13:7. doi: 10.1186/s13002-017-0135-1 (PMC5251319; doi:10.1186/s13002-017-0135-1)
Supplement: Additional file 1: — Surveyed Diseases and Model Diagnostics. (DOCX 669 kb) [file 13002_2017_135_MOESM1_ESM.docx]

**Additional file 1**

*Diseases Surveyed*

Diseases were chosen to reflect a spectrum of characteristics, including old and new diseases, those where effective traditional treatments were available, and those associated with high and low rates of mortality and morbidity. These inclusion criteria allowed us to control, somewhat, for the impact of disease characteristics on expertise and consensus.

**Table 1: Livestock Diseases in the Koore and Maasai that were included in the TVM questionnaire.**

| Koore | | Maasai | |
| --- | --- | --- | --- |
| *Korete* | Modern | *Maa* | Modern |
| Gandhi | *Trypanosomiasis* | Orkeipei | Contagious bovine pleuro-pnuemonia |
| Budhata | Black Leg | Orltikana | East Coast Fever |
| Teresa | ? | Olodokuolack | ? |
| Holsisa | ? | Ormilo | Bovine cerebral theileriosis |
| Bila | ? | Oltipilikwa | ? |
| Mannsah | Foot and Mouth Disease | Ndorobo | *Trypanosomiasis* |
| Irra Harge | ? | Olirobi | Foot and Mouth Disease |
| Funtse | Lumpy Skin Disease | Nunuk (Swahili) | Ephemeral Fever |
| Sheba | ? | Ndaragise | ? |
| Woransa | ? | Ohhlirrarerye | ? |

*Pharmaceutical List*

In surveying pharmaceutical information an empty bottle/satchet of the particular drug was shown to the interviewee given variations in local names. An empty syringe was used to verify the dosage amount. This was particularly helpful for children, who had observed their fathers administering drugs but were not aware of the cubic centimeter unit of measurement.

**Table 2. List of Pharmaceuticals included in VB questionnaire.**

| Koore | Maasai |
| --- | --- |
| Oxytetracycline (10%) (antibiotic) | Oxytetracycline (10%) (antibiotic) |
| Isometamidium chloride (anti-protozoa) | Alamais (20%) (antibiotic, brand name) |
| Penicillin (antibiotic) | Penicillin Streptomycin (antibiotic) |

**Regression Diagnostics and Model Fit**

Regression diagnostics included examination of outliers, collinearity, and normality and homogeneity of residual for each final model (i.e. Koore TVM; Koore VB; Maasai TVM; Maasai VB). Kernel density, standardized normal probabilities and normal quantile plots indicated some slight departures from normality (see Figures 1-Figure 12), particularly for models predicting expertise in veterinary biomedicine. To determine effects of these issues on estimation, we reran models excluding outlying studentized residual values exceeding +2 and -2 (see Table 1-4). In general, these models revealed no substantive differences when compared to the final models with the exception of two estimates. First, sex differences in TVM expertise within the Koore became non-significant (see Table 1) although the p-value was still trending towards significance (p=.068). Second, Maasai herd size became negatively correlated with VB expertise (see Table 3), although the effect size was very small β=.003, meaning that for every additional livestock unit the VB score decreased by .003 points. Residuals versus predicted value plots revealed some heteroscedasticity in the VB models, although the most extreme was for the Maasai, where no factors were significantly related (see Figures 13-16). Finally, all final models had Variance Inflation Factors below 3.0.

**Table 3. Koore TVM expertise with studentized residuals ±2 removed.**

|  | B | 95% C.I. |
| --- | --- | --- |
| Herd Size | 0.128 | 0.031 - 0.226* |
| VB Expertise | 0.756 | 0.380 - 1.133** |
| Sex | 5.951 | -0.470 - 12.372 |
| Age | 0.498 | 0.377 - 0.619** |
| Age Sqrd | -0.014 | -0.019 - -0.008** |
| Education | 0.454 | -1.273 - 2.181 |
| Income Types | 0.391 | -7.924 - 8.706 |
| Professional Help | -0.684 | -4.474 - 3.105 |
| Constant | 57.043 | 49.786 - 64.300** |
| Adjusted R2 | 0.62 |  |
| *N* | 73 |  |

**Table 4. Koore VB expertise with studentized residuals ±2 removed.**

|  | B | 95% C.I. |
| --- | --- | --- |
| Herd Size | 0.018 | -0.033 - 0.068 |
| TVM Expertise | 0.151 | 0.056 - 0.246** |
| Sex | -0.414 | -3.625 - 2.797 |
| Age | -0.026 | -0.107 - 0.055 |
| Age Sqrd | -0.002 | -0.007 - 0.004 |
| Education | -0.015 | -1.018 - 0.987 |
| Income Types | -0.156 | -4.335 - 4.023 |
| Professional Help | -0.837 | -2.780 - 1.107 |
| Constant | 97.025 | 93.234 - 100.817** |
| Adjusted R2 | 0.14 |  |
| *N* | 73 |  |

**Table 5. Maasai TVM expertise with studentized residuals ±2 removed.**

|  | B | 95% C.I. |
| --- | --- | --- |
| Herd Size | 0.001 | -0.002 - 0.003 |
| VB Expertise | -0.048 | -0.200 - 0.105 |
| Sex | 0.137 | -5.205 - 5.478 |
| Age | 0.407 | 0.285 - 0.529** |
| Age Sqrd | -0.007 | -0.011 - -0.002** |
| Education | -0.168 | -2.038 - 1.702 |
| Income Types | 1.914 | -0.844 - 4.673 |
| Professional Help | -0.910 | -2.929 - 1.109 |
| Constant | 81.330 | 75.871 - 86.790** |
| Adjusted R2 | 0.36 |  |
| *N* | 106 |  |

**Table 6. Maasai VB expertise with studentized residuals ±2 removed.**

|  | B | 95% C.I. |
| --- | --- | --- |
| Herd Size | -0.003 | -0.006 - -0.001** |
| TVM Expertise | 0.088 | -0.062 - 0.238 |
| Sex | 4.654 | -0.466 - 9.773 |
| Age | -0.037 | -0.165 - 0.090 |
| Age Sqrd | 0.005 | 0.001 - 0.010* |
| Education | 0.988 | -0.784 - 2.760 |
| Income Types | 2.209 | -0.479 - 4.897 |
| Professional Help | -1.040 | -2.966 - 0.886 |
| Constant | 76.968 | 71.583 - 82.352** |
| Adjusted R2 | 0.12 |  |
| *N* | 110 |  |

**Figure 1. Koore TVM:Kernel Density Plot.**


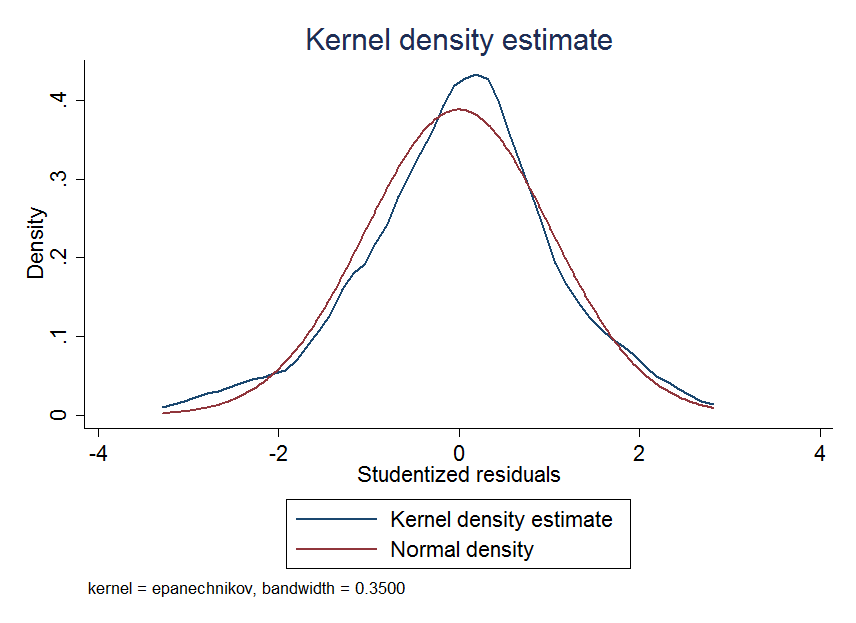


**Figure 2. Koore VB:Kernel Density Plot.**

**
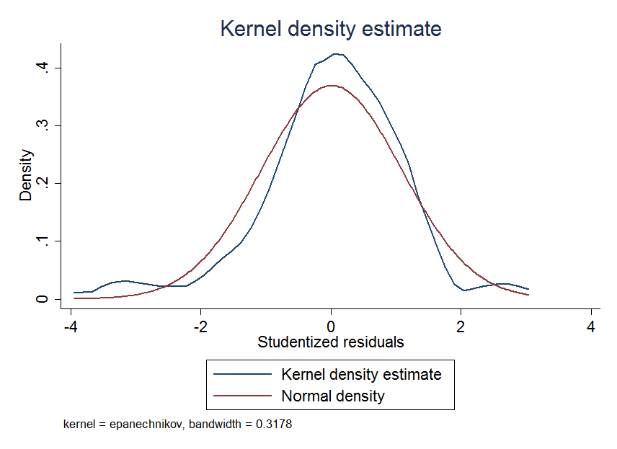
**

**Figure 3. Maasai TVM: Kernel Density Plot.**

**
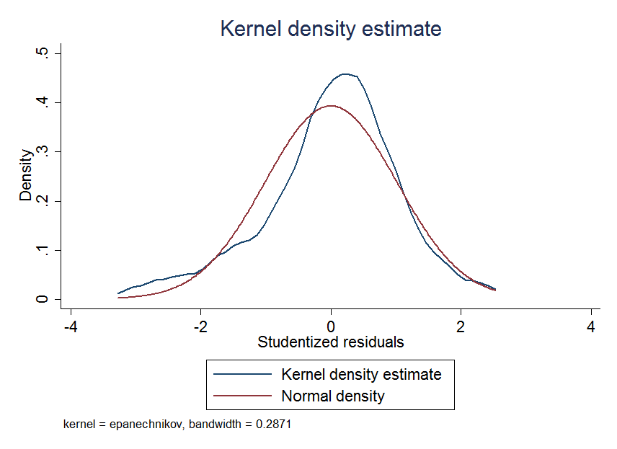
**

**Figure 4. Maasai VB: Kernel Density Plot.**

**
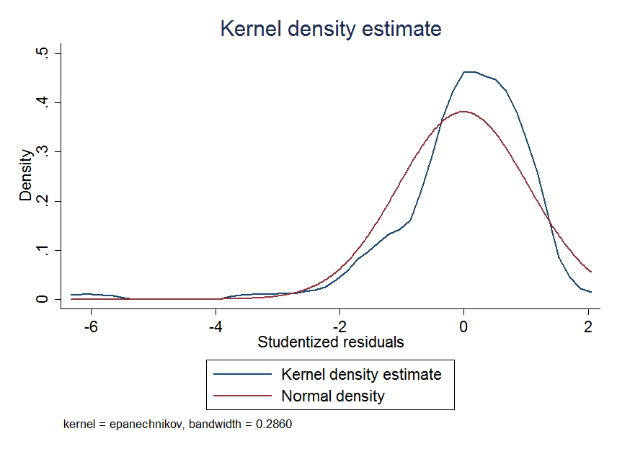
**

**Figure 5. Koore TVM: Standardized Normal Probability Plot.**

**3
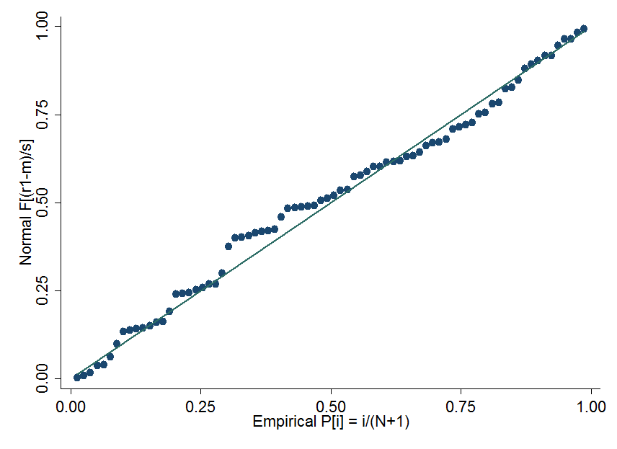
**

**Figure 6. Koore TVM: Normal Quantile Plot.**

**
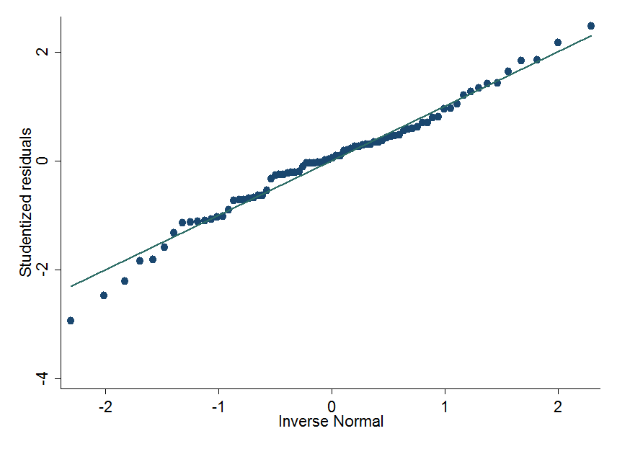
**

**Figure 7. Koore VB: Standardized Normal Probability Plot.**

**
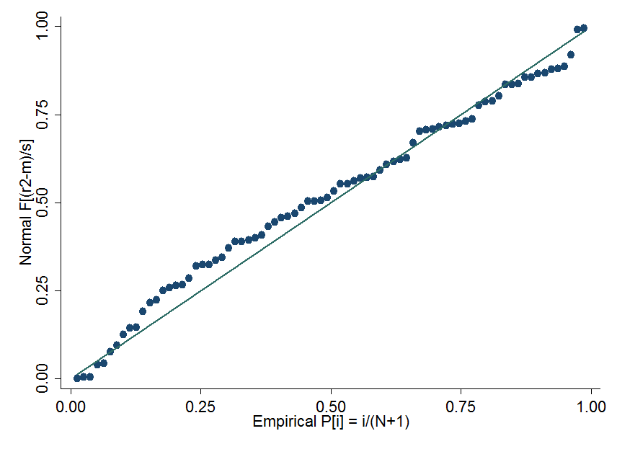
**

**Figure 8. Koore VB: Normal Quantile Plot.**

**
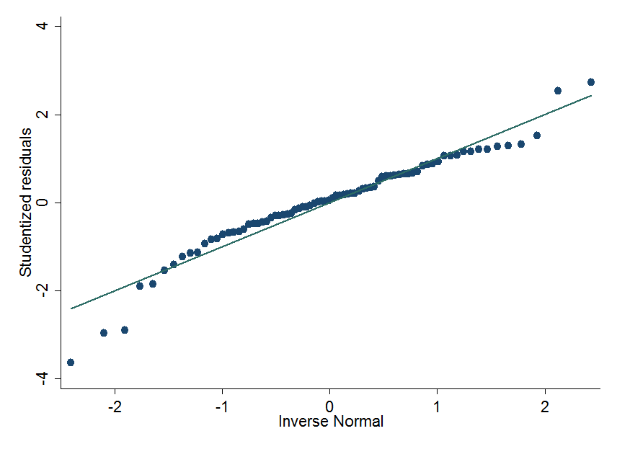
**

**Figure 9: Maasai TVM: Standardized Normal Probability Plot.**

**
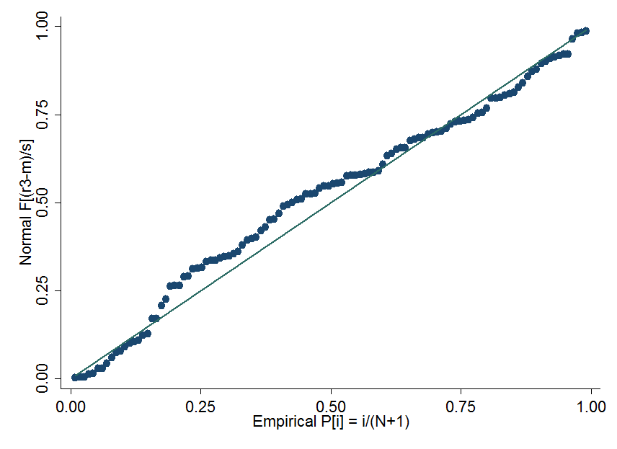
**

**Figure 10. Maasai TVM: Normal Quantile Plot.**

**
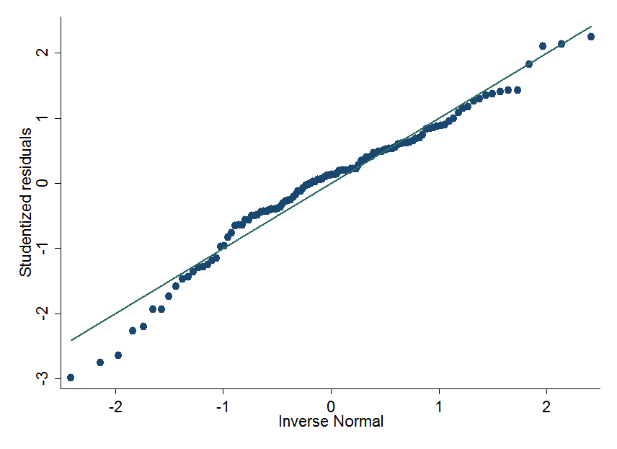
**

**Figure 11. Maasai VB: Standardized Normal Probability Plot.**

**
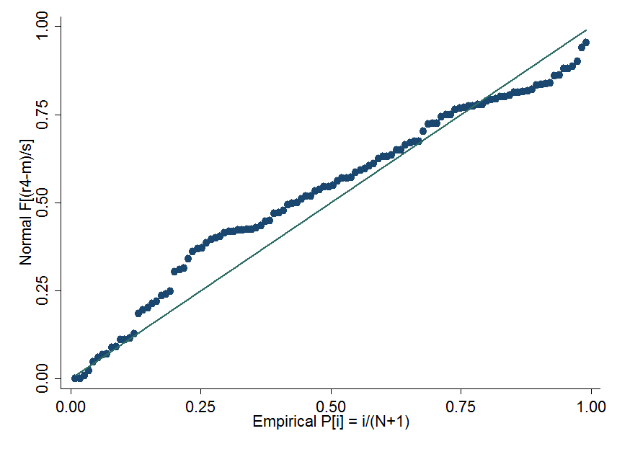
**

**Figure 12. Maasai VB. Normal Quantile Plot.**

**
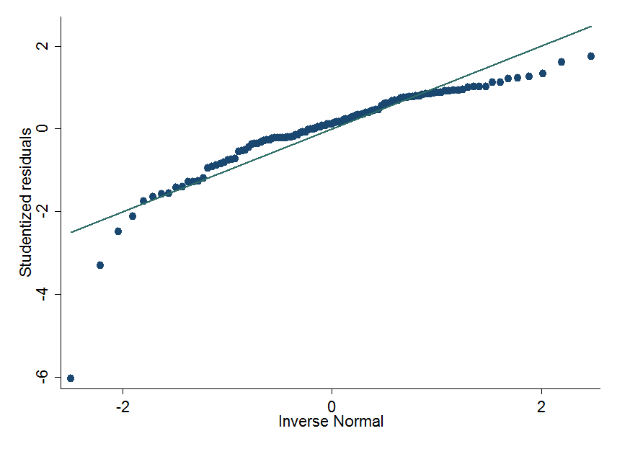
**

**Figure 13. Koore TVM: Residual versus Predicted Plot.**

**
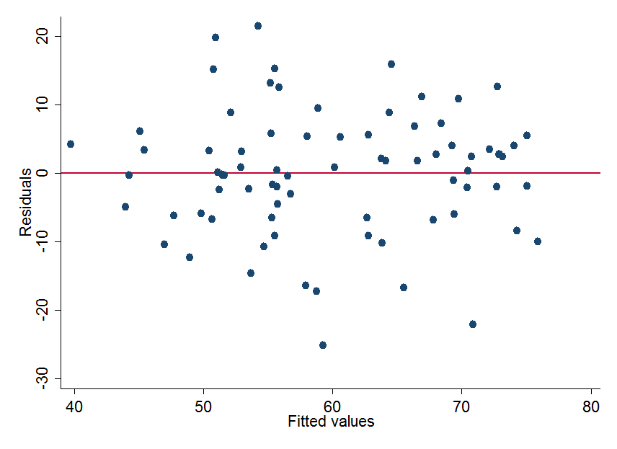
**

**Figure 14. Koore VB: Residual versus Predicted Plot.**

**
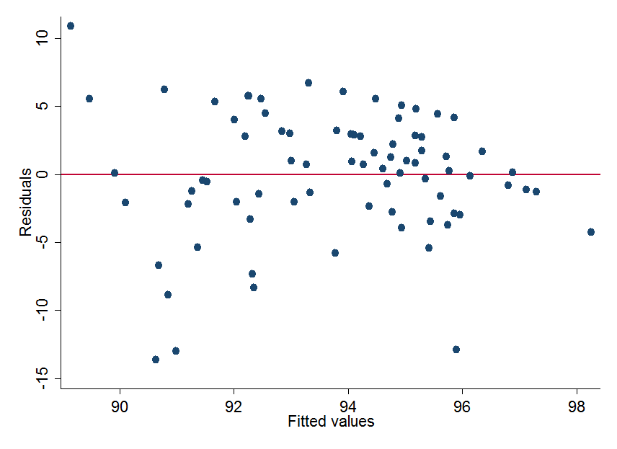
**

**Figure 15. Maasai TVM: Residual versus Predicted Plot.**

**
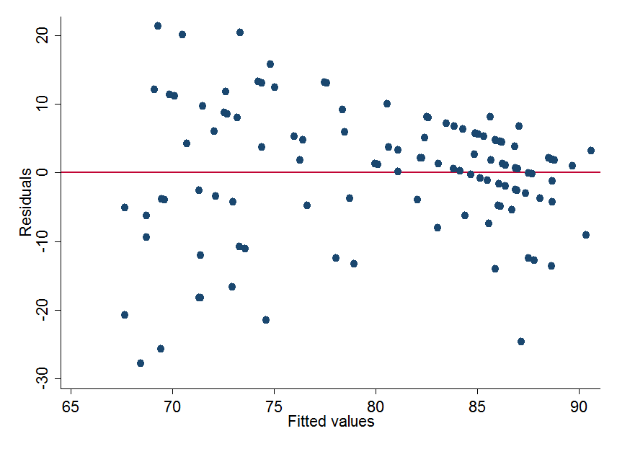
**

**Figure 16. Maasai VB: Residual versus Predicted Plot.**

**
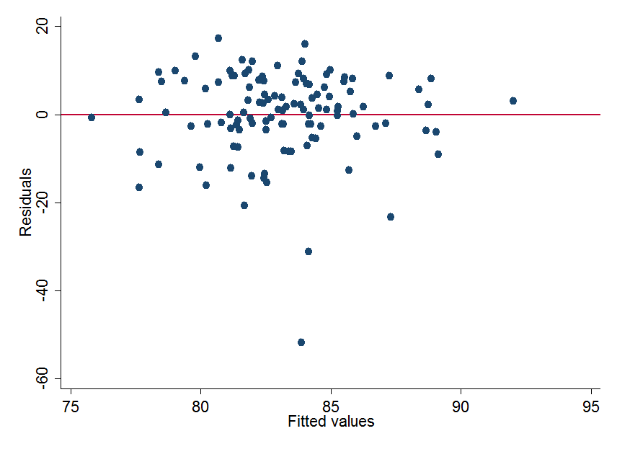
**
